# Low-Dose Systemic Thrombolysis as Rescue Therapy After Catheter-Directed Thrombectomy for High-Risk Pulmonary Embolism on VA-ECMO

**DOI:** 10.1016/j.jaccas.2026.107354

**Published:** 2026-03-14

**Authors:** Shamali Nehete, Aditi Kothari, Leo C. Mercer, Abdur R. Jamal, Nils P. Nickel

**Affiliations:** a Department of Internal Medicine, Texas Tech University Health Sciences Center El Paso, El Paso, Texas, USA; b Division of Trauma and Acute Care Surgery, Texas Tech University Health Sciences Center El Paso, El Paso, Texas, USA; c Division of Vascular Surgery, Texas Tech University Health Sciences Center El Paso, El Paso, Texas, USA; d Division of Pulmonary and Critical Care Medicine, Texas Tech University Health Sciences Center El Paso, El Paso, Texas, USA

**Keywords:** pulmonary circulation, pulmonary embolism, right heart failure, VA-ECMO

## Abstract

**Background:**

Catheter-directed treatments (CDTs) are increasingly being used for high-risk pulmonary embolism (PE) instead of upfront systemic thrombolysis.

**Case Summary:**

A patient with massive PE presented with hemodynamic instability and right ventricular (RV) strain after 2 thrombectomies. Low-dose systemic thrombolysis was initiated while the patient was on venoarterial extracorporeal membrane oxygenation (VA-ECMO), which led to improved perfusion and gas exchange.

**Discussion:**

This case highlights that systemic thrombolysis may offer advantages over CDT by providing global fibrinolysis, which can address diffuse distal clot burden and microvascular obstruction that focal CDTs may not fully treat.

**Take-Home Messages:**

In high-risk PE with shock and contraindications or failure of CDT, systemic thrombolysis remains the first-line reperfusion therapy. This case demonstrates that low-dose systemic alteplase can be safely administered on venoarterial extracorporeal membrane oxygenation to restore reperfusion and unload the failing right ventricular, leading to resolution of shock.

## Visual Summary

**Figure:**
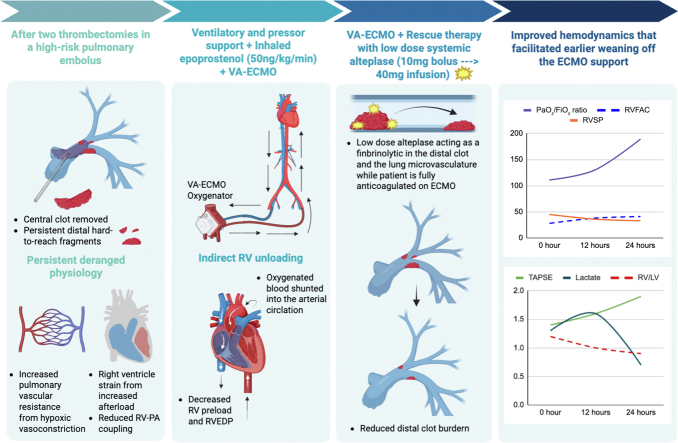

**Visual Summary:**
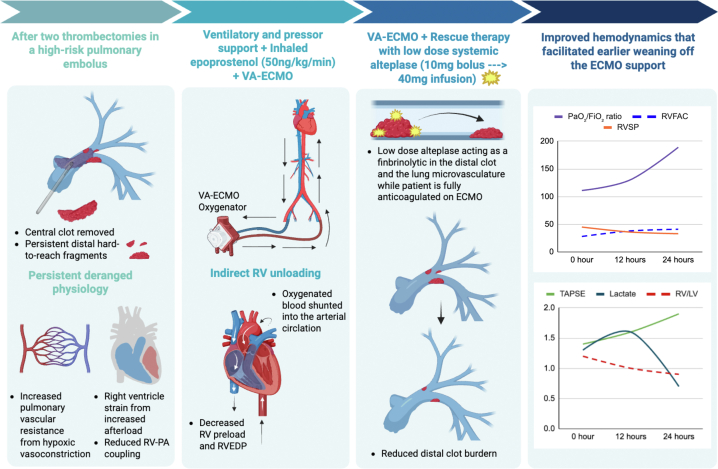
Sequence of Events After Failed Catheter-Directed Thrombectomy Followed by Low-Dose Systemic Thrombolysis as Rescue Therapy on VA-ECMO Created in BioRender, Nehete S. (2026), https://BioRender.com/aa4jehz PaO2/FiO2 = arterial oxygen partial pressure to fraction of inspired oxygen ratio; RVEDP = right ventricle end-diastolic pressure (mmHg); RVFAC = right ventricular fractional area change (%); RV/LV = right-to-left ventricular end-diastolic diameter ratio by echocardiography; RV-PA = right ventricle-pulmonary artery; RVSP = right ventricular systolic pressure (mmHg); TAPSE = tricuspid annular plane systolic excursion (cm); VA-ECMO = venoarterial extracorporeal membrane oxygenator.

## History of Presentation

A 28-year-old man with no known past medical history presented to an outside hospital with dyspnea, chest, and back pain, and was found to have a saddle pulmonary embolism (PE) with right ventricular (RV) strain. On admission, he was febrile with a heart rate of 137 beats/min, shock index 1.27, respiratory rate 26 breaths/min, blood pressure 108/78 mm Hg, and oxygen saturation 98% on room air.

**Take-Home Messages**

• In massive (high-risk) pulmonary embolism with shock and contraindications or failure of catheter-directed treatment, systemic thrombolysis remains the guideline-supported first-line reperfusion option when bleeding risk is acceptable.
• This case demonstrates that low-dose systemic alteplase can be safely administered on venoarterial extracorporeal membrane oxygenation to restore pulmonary reperfusion and rapidly unload the failing right ventricle, leading to resolution of shock and end-organ dysfunction.

## Investigations

**Figure 1:**
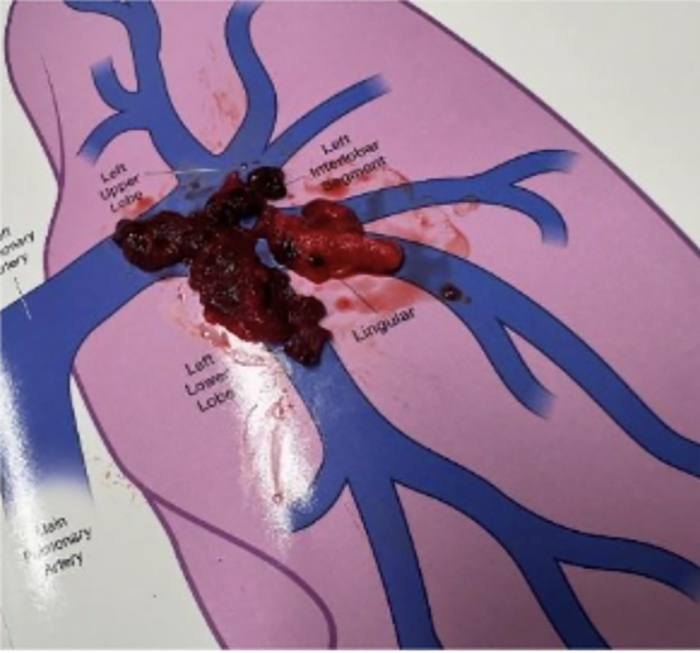
Gross Thrombus Specimen Retrieved During the Second CDT Gross thrombus fragments retrieved during aspiration thrombectomy from the left lung, shown on an anatomic pulmonary arterial diagram for orientation. CDT = catheter-directed treatment.

**Figure 2:**
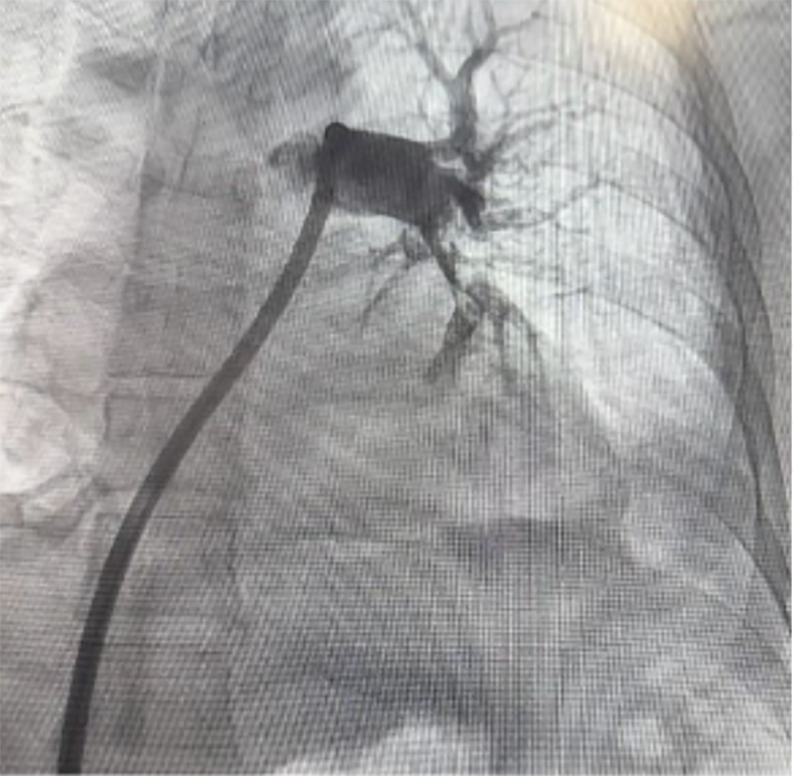
Pulmonary Angiography After Second CDT Selective left lung pulmonary angiography performed during catheter-directed aspiration thrombectomy, demonstrating contrast opacification of lobar and segmental pulmonary arterial branches with persistent irregularity/filling defects consistent with residual thrombus and incomplete reperfusion. CDT = catheter-directed treatment.

Initial laboratory studies were notable for creatinine 1.2 mg/dL, lactate 1.3 mmol/L, hs-troponin 1,151.2 ng/L, and B-type natriuretic peptide 235 pg/mL. COVID-19 was ruled out by a polymerase chain reaction–based nasal swab. No other causes for infection were found. The fever was believed to be related to the acute venous thromboembolism, particularly in the setting of a massive PE. He was started on an intravenous heparin infusion and underwent a catheter-directed treatment (CDT) using the Penumbra Lightning Flash 16-F catheter for aspiration thrombectomy. A repeat computed tomography pulmonary angiogram following the procedure demonstrated persistent pulmonary emboli with ongoing RV strain, and a second CDT using the same catheter was performed 24 hours later. Gross examination of the removed thrombi shows multiple proximal fragments retrieved during the second CTD, displayed on an anatomic pulmonary arterial diagram for orientation (Figure 1). Despite large proximal clot removal, a selective pulmonary angiography performed during the second CDT demonstrates distal opacification of lobar and segmental branches with persistent irregularity/filling defects consistent with residual thrombus in more distal branches of the pulmonary arteries (Figure 2). After the second intervention, the patient acutely deteriorated with hypotension and respiratory distress, requiring endotracheal intubation, initiation of vasopressors, and transfer to our center for consideration of extracorporeal support.

## Management

On arrival at our hospital, he was intubated and mechanically ventilated, with a blood pressure of 96/65 mm Hg while receiving norepinephrine and vasopressin. Point-of-care ultrasound revealed a severely dilated right ventricle based on an elevated right-to-left ventricular end-diastolic diameter ratio by echocardiography (1.2), with reduced systolic function, based on reduced tricuspid annular plane systolic excursion (1.4 cm) and right ventricular fractional area change (28%). The left ventricle appeared hyperdynamic and underfilled. The patient was also hypoxemic with an arterial oxygen partial pressure to fraction of inspired oxygen ratio of 111, which is a global measure of oxygenation, with a ratio >300 considered as normal and a ratio <100 indicating severe hypoxemia.

There was no noticeable clinical improvement in his hemodynamics or hypoxia after initiation of inhaled epoprostenol at 50 ng/kg/min, and the decision was made to cannulate for venoarterial extracorporeal membrane oxygenation (VA-ECMO) to stabilize his hemodynamics and to allow for RV-protective ventilation. After VA-ECMO cannulation and hemodynamic improvement, a repeat computed tomography pulmonary angiogram (on reduced ECMO flows) demonstrated persistent bilateral occlusive proximal pulmonary emboli with RV strain. In addition, lower extremity venous duplex ultrasound revealed nonocclusive thrombus in the left femoral, popliteal, posterior tibial, and peroneal veins.

Given the degree of persistent clot burden, RV and hypoxic failure, the decision was made to treat the patient with a low-dose alteplase protocol to minimize bleeding risk while on full anticoagulation and on VA-ECMO. He received a 10 mg alteplase bolus, followed by an infusion of 40 mg over 6 hours on day 1 of VA-ECMO.

## Outcome and Follow-Up

Within 12 hours after alteplase, the patient showed significant improvements in hemodynamics and gas exchange, vasopressors were weaned, and his arterial oxygen partial pressure to fraction of inspired oxygen ratio improved from 111 to 200. The patient passed a VA-ECMO weaning and was successfully decannulated 2 days after the initiation of VA-ECMO. The patient was transferred back 7 days after VA-ECMO decannulation to the referring hospital, from where he was discharged home after 6 days without supplemental O_2_ and in good functional class. A hypercoagulability work-up was initiated at the referring outside hospital, to which the patient ultimately returned after clinical improvement and transfer from the intensive care unit to the medical ward. Outpatient hematology and pulmonary follow-ups were also arranged. Because the work-up was performed at the outside facility after transfer back, we do not have access to the complete results.

## Discussion

PE is a clinical syndrome with a broad spectrum of severity, ranging from incidental low-risk events with excellent survival to high-risk PE with high mortality. In some cases, it also represents a continuum of right-ventricular involvement—from compensated RV pressure overload with preserved perfusion to progressive RV dysfunction and decompensation with hemodynamic instability—highlighting the importance of early risk stratification and reassessment.^1^^,^^2^ Multiple international guidelines—including those from the American Heart Association, American College of Chest Physicians (CHEST), European Society of Cardiology and European Respiratory Medicine, the Pulmonary Embolism Response Team Consortium, the American Society of Hematology, and the UK's National Institute for Health and Care Excellence—converge on the recommendation that systemic pharmacologic thrombolysis should be considered first-line reperfusion therapy in patients with high-risk (massive) PE who do not have prohibitive bleeding risk.^3^

However, with the increasing popularity of catheter-directed thrombectomy, an increasing number of patients with PE and impending hemodynamic instability are not treated with upfront systemic thrombolysis.^4^ The growing “thrombectomy-first” practice pattern is driven largely by concerns about bleeding, the perception of better hemodynamic control, and device availability rather than by definitive comparative outcome data. Adequately powered randomized trials directly comparing CDT with systemic thrombolysis are still lacking. This shift highlights the difficulty of clinical management of PE patients with progressive hemodynamic instability.

Here, we report, to the best of our knowledge, the first case of low-dose alteplase on VA-ECMO as rescue therapy after 2 failed CTD attempts. VA-ECMO bypasses the failing RV by pulling the blood away from the central venous system and displacing oxygenated blood directly into the arterial circulation, thereby reducing the RV strain by lowering the preload and RV end-diastolic pressures. Some case reports describe successful thrombectomy on VA-ECMO for high-risk PE.^5^^,^^6^ However, several features can limit the effectiveness of CDT in truly high-risk PE. Large bilateral proximal clot burden, especially when centered at the main pulmonary artery bifurcation (saddle/near-saddle morphology), can exceed the effective “purchase” and aspiration efficiency of some devices, leaving substantial residual thrombus despite multiple passes.^7^ Our case suggests that systemic thrombolysis may in fact confer advantages over catheter-directed therapy by providing global fibrinolysis, thereby targeting diffuse distal thrombus within the pulmonary resistance vasculature—an important determinant of pulmonary vascular resistance and RV afterload—and potentially improving pulmonary capillary perfusion and gas exchange.^8^ VA-ECMO by lowering the RV preload and systemic thrombolysis by reducing the RV afterload enhanced the right ventricle-pulmonary artery coupling, which is a crucial determining factor for successful ECMO weaning.

Given an elevated bleeding risk with systemic thrombolysis on VA ECMO,^9^ we decided to pursue low-dose systemic alteplase (10 mg bolus, 40 mg over 6 hours), because our center has had positive experience with this regimen for PE and associated RV failure.^10^ Under VA-ECMO support, this regimen led to rapid improvement in hemodynamics and gas exchange, enabling successfully decannulation within 48 hours (see Table 1). The initial tissue plasminogen activator bolus is intended to initiate early and rapid fibrinolytic activity at a critical moment of hemodynamic vulnerability, whereas the short, fixed-duration infusion is aimed to balance timely reperfusion against cumulative bleeding exposure—particularly relevant on VA-ECMO and after multiple days of therapeutic anticoagulation. Conceptually, this approach prioritizes early RV afterload reduction and restoration of pulmonary perfusion (improving both hemodynamics and gas exchange) while preserving the ability to stop the infusion promptly if bleeding emerges and to reassess response quickly under ECMO support.

**Table 1:** Serial Cardiopulmonary Parameters Before and After Low-Dose Systemic Alteplase Administered During Venoarterial Extracorporeal Membrane Oxygenation Support

	PaO_2_/FiO_2_ Ratio	Lactate	RV/LV	TAPSE	RVSP	RVFAC
Admission	111	1.3	1.2	1.4	45	28
Post-alteplase (12 h)	130	1.6	1.0	1.6	36	38
Post-alteplase (24 h)	189	0.7	0.9	1.9	33	41

Admission values were obtained on arrival to the receiving intensive care unit before alteplase administration; post-tPA values were recorded 12 hours and 24 hours after initiation of the alteplase regimen.

PaO_2_/FiO_2_ ratio = arterial oxygen partial pressure to fraction of inspired oxygen ratio; RV/LV = right-to-left ventricular end-diastolic diameter ratio by echocardiography; RVFAC = right ventricular fractional area change; RVSP = estimated right ventricular systolic pressure; TAPSE = tricuspid annular plane systolic excursion; tPA = tissue plasminogen activator.

## Conclusions

This case illustrates that in high-risk PE with persistent clot burden, and severe RV strain, and hypoxic respiratory failure after 2 unsuccessful CDT attempts, rescue low-dose systemic alteplase on VA-ECMO can achieve rapid reperfusion with meaningful hemodynamic and gas-exchange recovery, enabling early decannulation. Although thrombectomy offers an attractive “localized” strategy, in some cases, it may be insufficient and cannot reliably address distal thrombus and microvascular obstruction that sustain pulmonary vascular resistance and RV afterload. In the absence of definitive comparative outcomes data, a thrombectomy-first approach should not be presumed to replace systemic thrombolysis in high-risk PE.

Future prospective studies should define patient- and anatomy-specific criteria to guide optimal selection and sequencing of CDT vs systemic thrombolysis (including low-dose regimens), particularly in high-risk PE patients with right ventricular and respiratory failure.

## Funding Support and Author Disclosures

The authors have reported that they have no relationships relevant to the contents of this paper to disclose.
